# Lambert-Eaton Myasthenic Syndrome; Pathogenesis, Diagnosis, and Therapy

**DOI:** 10.4061/2011/973808

**Published:** 2011-09-29

**Authors:** Nils Erik Gilhus

**Affiliations:** ^1^Department of Clinical Medicine, University of Bergen, 5020 Bergen, Norway; ^2^Department of Neurology, Haukeland University Hospital, 5021 Bergen, Norway

## Abstract

Lambert-Eaton Myasthenic Syndrome (LEMS) is a rare disease with a well-characterized pathogenesis. In 50% of the patients, LEMS is a paraneoplastic manifestation and caused by a small cell lung carcinoma (SCLC). Both LEMS patients with SCLC and those without this tumour have in 85% of cases pathogenetic antibodies of very high LEMS specificity against voltage-gated calcium channels (VGCCs) in the cell membrane of the presynaptic motor nerve terminal. Better understanding of LEMS pathogenesis has lead to targeted symptomatic therapy aimed at the neuromuscular junction and to semispecific immuno-suppression. For SCLC LEMS, tumour therapy is essential.

## 1. Introduction

The neuromuscular synapse represents a predilection site for disease. Autoimmune, genetic, and toxic disorders are linked to the neuromuscular junction. The dominating symptom of all such disorders is muscular weakness. The disorders interfere with the acetylcholine-mediated transmission of the signal from the presynaptic nerve to skeletal muscles, impairing muscle contraction. Both the autoimmune, genetic and toxic conditions can effect either pre- or postsynaptically. Mutated genes leading to a change in protein function result in myasthenic syndromes of various types, the postsynaptic acetylcholine receptor most often the target, or also proteins in the postsynaptic membrane functionally linked to this receptor. Toxins exert their function pre- or postsynaptically and will paralyze either attacker or prey in nature's fight for survival. Such toxins are widely used in medicine, both therapeutically and for diagnostic and research purposes. 

Lambert-Eaton Myasthenic Syndrome (LEMS) represents one of the distinct autoimmune disorders at the neuromuscular junction. In 1956, Lambert and coworkers reported 6 patients with atypical myasthenia, lung carcinoma, and a specific response to repeated nerve stimulation differing from myasthenia gravis [1]. During recent years, disease mechanisms have been thoroughly elucidated for LEMS, so that this disorder can now be characterized as a model disease for other autoimmune and paraneoplastic disorders. LEMS is caused by pathogenic autoantibodies to presynaptic voltage-gated calcium channels (VGCCs) in the membrane of the motor nerve terminal, impairing acetylcholine release, and thereby causing distinct weakness of striated skeletal muscles. The challenge now is to transfer this detailed pathogenetic knowledge into even more effective therapy.

## 2. Epidemiology

LEMS fulfils the criteria for a rare disease. In a study from South Holland, Wirtz et al. [2] found a LEMS prevalence of 2.3 per million and an annual incidence rate of 0.5 per million. This incidence was 1.4 times lower than what they found for myasthenia gravis. A low prevalence relative to incidence reflects the poor survival of LEMS patients with the paraneoplastic type of disease. 60% of the LEMS patients were males. Mean age of debut was 58 years. There seems to be two peaks for age of onset, one around 40 years and one at a higher age, similar to what is seen for myasthenia gravis [3].

LEMS is subclassified into two main subgroups; LEMS combined with small cell lung carcinoma (SCLC), and LEMS with no SCLC. The no-SCLC LEMS group is dominating regarding prevalence as this group has a near normal survival rate. No-SCLC LEMS patients have a lower age of debut than SCLC LEMS [3, 4]. LEMS with SCLC shows a male preponderance, reflecting smoking habits. The frequency of LEMS among the total SCLC patient population is reported between 0.5 and 3% [2, 5]. LEMS-related autoantibodies occur in a higher proportion of SCLC patients, but without leading to manifest neuromuscular disease. SCLC patients with LEMS tend to be younger than those without LEMS [6].

## 3. Clinical Picture

Muscle weakness represents the hallmark of LEMS. This weakness starts nearly always in proximal muscle groups, especially in the legs. 80% of LEMS patients experience proximal weakness in both arms and legs [4, 7, 8]. Also facial weakness, eye muscle complaints, bulbar muscular weakness, and distal pareses are relatively common. LEMS with SCLC tends to have more severe muscle weakness and with a distinct progression. Areflexia is a common finding. 

Autonomic dysfunction is the second typical symptom of LEMS. Such symptoms are milder and have less functional significance than muscular weakness. However, it affects a large majority of LEMS patients. Dry mouth, dry eyes, erectile dysfunction, constipation and reduced sweating are frequently confirmed when examining LEMS patients, and to the same degree for patients with and without SCLC.

## 4. Pathogenesis

LEMS is caused by autoantibodies to VGCC in the presynaptic neuronal cell membrane. Such antibodies show a high sensitivity, as they can be detected in 85% of all LEMS patients. The LEMS specificity in patients with distinct muscle weakness is nearly 100%. Among SCLC patients without any symptoms of muscle weakness or autonomic dysfunction, 3–5% have VGCC antibodies. VCCC antibodies are hardly ever found in other control groups, but have been described in patients with clinically pure cerebellar ataxia.

The presynaptic release of acetylcholine is a complex process. The VGCC antibodies in LEMS lead to a reduction in the quantal release of acetylcholine [9]. A direct pathogenetic effect of the autoantibodies has been shown by injection in experimental animals and supported by the patients' clinical and electrophysiological response to plasma exchange with removal of the autoantibodies. The number of VGCC is reduced in LEMS patients, caused by antibody-mediated cross-linking of the ionic channels. Research groups in Oxford and at the Mayo Clinic have been very active in elucidating these disease mechanisms. 

The autonomic dysfunction in LEMS is probably caused by the same VGCC antibodies that cause the muscle weakness. The antibodies impair transmitter release from parasympathetic and sympathetic neurons through downregulation of the receptors [10, 11].

VGCC mediates calcium influx into the nerve terminal. This influx activates presynaptic signalling pathways. Synaptotagmin, synaptobrevin, synthaxin, and SNAP-25 are molecules taking part in the interaction between the increased intracellular calcium concentration and the release of acetylcholine from preformed synaptic vesicles. With calcium influx being hampered by the VGCC antibodies, presynaptic compensatory mechanisms influence acetylcholine release. This complex interaction has recently been reviewed by Takamori [11]. Non-VGCC molecules influencing presynaptic acetylcholine release have been examined as potential targets for autoantibodies in LEMS patients without VGCC antibodies. By a similar approach, MuSK was identified as an alternative antigen target in myasthenia gravis. For LEMS, alternative antigens have been suggested but not finally proven

VGCC represents multisubunit ionic channels, and comprising 4 or 5 subunits. Membrane depolarisation opens the central pore for calcium influx. Electrical signals are thereby coupled to neurotransmission, secretion, and other events in various cell types. Nonvoltage-gated calcium channels respond to other types of stimuli, for example mechanical stretch. The role of VGCC for neuromuscular synaptic transmission and disease was recently nicely reviewed by Urbano et al. [12], that review concentrating on the molecular processes related to VGCC subtypes. VGCC were initially grouped according to tissue where they were detected, and/or their pharmacological properties; L, P/Q, N, K, T. The LEMS autoantibodies are directed selectively against the P/Q subtype of VGCC. More recently VGCC has been grouped in an alternative way according to the gene name of their alpha 1 subunit, which also reflects their protein structure. VGCC properties have now been examined in detail and linked to molecular sequence. The VGCC recognized by the autoantibodies in LEMS are of the Ca_v_ 2.1 subtype, both at the motor and autonomic axon terminals. The antibodies may to some degree bind also to other VGCC subtypes, especially to Ca_v_ 2.2 [13]. However, the primary target and the cause of the down-regulation is binding to Ca_v_ 2.1 [12, 14]. Although VGCC downregulation represents the main mechanism for dysfunction, also a direct antibody-mediated channel block has been reported. Most VGCC antibodies in LEMS are directed against the alpha-1 subunit. The exact pattern of epitope reactivity seems to differ between LEMS patients with and without SCLC [15].

The role of T lymphocytes has not been established in LEMS. T cells do not aggregate around the presynaptic terminal. In contrast to myasthenia gravis, no morphological or functional disturbances have been reported in thymus or other lymphoid organs. However, the expression of T cell markers in LEMS patients suggested a down-regulation of immunosuppression in SCLC patients with LEMS, compared to such patients without LEMS [16]. T cell immunoregulation may therefore facilitate or counteract the development of LEMS. T cell activity in the SCLC tissue may be relevant for the induction of LEMS. 

A genetic susceptibility has been established for nearly all autoimmune disorders, both from family history and from susceptibility genes, especially HLA-antigens. LEMS without SCLC is significantly associated with HLA-B8 (HLA- class I), and HLA -DR3 and -DQ2 (HLA-class II) [3, 17]. About two-thirds of nontumour LEMS patients compared to one-third of controls have this HLA-pattern. This is not surprising, as the same HLA genotypes are found with increased frequency in most autoimmune disorders, including myasthenia gravis. The clinically well-known autoimmune overlap manifests through this joint genotype. A unique observation is the report of monozygous twins, one with LEMS and VGCC antibodies, the other with myasthenia gravis and acetylcholine receptor antibodies [18]. 

In contrast, no relation has been found for SCLC LEMS and HLA [3]. This indicates a pathogenetic difference between the two LEMS subtypes. The same observation is true for myasthenia gravis, where there is no HLA-association for the paraneoplastic, thymoma-associated subtype. Nor do paraneoplastic disorders in general show a consistent HLA-pattern. Patients with paraneoplastic disorders do not have an increased frequency of the HLA-genotypes that are associated with nonparaneoplastic autoimmunity. Tumour tissue from SCLC LEMS patients expresses a reduced amount of HLA class I antigens compared to tissue from SCLC patients without LEMS [19].

For the 50% of LEMS patients with a SCLC, the tumour represents the initiating LEMS event. VGCC are expressed on the surface of the SCLC cells [20]. This expression of cancer-related neoantigens induces the autoantibody production, and the autoantibodies cross-react with presynaptic VGCC antigens. This induction of autoimmunity usually takes place early in tumour development, in most patients before a SCLC diagnosis has been established, and before even a malignant or lung disease has been suspected. 

In the remaining LEMS patients, that is, those with no SCLC, no initiating event can be identified. Such patients do not develop a SCLC or any another malignant or lung disorder later, linked to their LEMS. This is again similar to other autoimmune disorders. Cross-reactivity of antibodies occurring as a response to a clinical or subclinical infection would have been a plausible explanation, but has been impossible to confirm. 

Antibodies against SOX proteins (sry-like high-mobility group box) represent a specific serological marker for SCLC [6]. No pathogenetic role has been established for the SOX antibodies. However, they occur more frequently in SCLC LEMS (67%) than in SCLC no-LEMS (36%).

## 5. Diagnosis

The LEMS diagnosis is suspected from typical clinical symptoms; the triad of muscle weakness with a typical distribution, areflexia, and autonomic dysfunction. Presence of VGCC autoantibodies confirms the LEMS diagnosis, due to the very high antibody specificity. Absence of detectable VGCC antibodies does not rule out LEMS. Neurophysiological tests with adequate repetitive stimulation undertaken in relevant muscles strongly support a diagnosis of LEMS. Therapeutic response to drugs increasing acetylcholine availability at the postsynaptic receptor is expected, but has no strong diagnostic value, less than for myasthenia gravis. The most frequent misdiagnosis is probably seronegative and atypical myasthenia gravis or unspecified myasthenic syndromes.

Once a diagnosis of LEMS has been confirmed, or even suspected, starts the search for a SCLC. Smoking markedly increases this risk, but nonsmokers should undergo the same diagnostic program. Extensive imaging is necessary, and if necessary including PET. If the initial search is negative, the screening should be repeated after 3 months, and then every 6 months up till 2 years after LEMS debut, this according to recent EFNS guidelines [21]. SOX antibodies represent an additional marker of diagnostic value in LEMS patients, as they have high specificity for SCLC, although lower sensitivity [6]. Two national cohorts (Dutch and English) with a total of 219 patients were recently used to develop a clinical score for predicting SCLC in LEMS [22]. Age at onset, smoking, weight loss, general well-being, bulbar involvement, male sexual impotence, and SOX antibodies were all independent predictors for SCLC in LEMS.

## 6. Associated Disease

The most important disease association is the one between LEMS and SCLC. One half of LEMS patients have a paraneoplastic disorder. Other tumours do probably not occur with any increased frequency in LEMS. Whereas SCLC can be linked to various paraneoplastic disorders and various autoantibodies, LEMS is linked to a SCLC only.

LEMS patients with no SCLC have an increased occurrence of other autoimmune disorders, at least in part due to a genetic predisposition for autoimmune reactivity.

An increasing number of rare disorders of the central nervous system have been found to be associated with serum autoantibodies to ion channels, receptors, or associated proteins in the cell membrane. Some of these autoantibodies are also pathogenic. Encephalitis can be caused by antibodies to voltage-gated potassium channels (VGKC) and to NMDA-receptors, and antibodies to GAD and aquaporin 4 are associated with distinct neurological syndromes. VGCC antibodies and LEMS can also coexist with central nervous system disease. Among SCLC patients diagnosed with paraneoplastic subacute cerebellar degeneration, 16% were found with concomitant LEMS, and 24% with increased VGCC antibody levels [23, 24]. Cerebellar ataxia has been reported also in a few nonparaneoplastic LEMS patients with VGCC antibodies [25].

## 7. Therapeutic Principles

LEMS pathogenesis points directly to potential treatment principles. The reduced quantal content release of acetylcholine can be counteracted by symptomatic therapy. Acetylcholine esterase inhibition will increase the amount of acetylcholine in the synaptic cleft. Therapy with pyridostigmine and similar inhibitors has usually a positive effect, but less so and less predictably than in myasthenia gravis. Acetylcholine esterase inhibition should be tried, but is perhaps not first-line therapy [26, 27]. 3,4 diaminopyridine is an aminopyridine that blocks presynaptic voltage-gated potassium channels and thereby prolongs the duration of the presynaptic action potential. The amount of acetylcholine released increases. The positive clinical effect of 3,4 diaminopyridine is well documented [26, 28]. The 3,4 diaminopyridine phosphate salt has recently been marketed [9]. 

If symptomatic LEMS treatment is insufficient, immunosuppressive drug therapy should be initiated. A combination of prednisone/prednisolone and azathioprine is best documented [26, 29, 30]. For other immunosuppressive drugs, there are mostly limited series and case reports published. Mycophenolate and cyclosporine have been recommended, probably also because they are used for myasthenia gravis. Rituximab is a monoclonal antibody that specifically targets B lymphocytes. This drug should therefore be promising for all autoantibody-mediated disorders, including LEMS. A positive treatment result in a few patients has recently been reported [28, 31]. Intravenous immunoglobulin is used for several paraneoplastic disorders, and it has a well-proven effect for acute exacerbations of myasthenia gravis as well. A beneficial short-term effect has been reported for LEMS, probably to the same degree for paraneoplastic and no-tumour LEMS. An EFNS guideline concludes that intravenous immunoglobulin may be tried in LEMS [32]. Plasma exchange has probably a similar effect, but is less useful as a long-term therapy [30].

Physical training can be carried out safely in mild and moderate LEMS [26]. Overweight should be avoided. All complicating disorders, such as respiratory infections, should be vigorously treated. Drugs with a potential negative impact on neuromuscular transmission should be avoided. Standard vaccination programmes are recommended also for LEMS patients. Treatment with intravenous immunoglobulin during pregnancy should be considered due to the risk of fetal arthrogryposis, similar to what is seen in myasthenia gravis. Transient neonatal LEMS due to transplacental transfer of IgG antibodies has been described [33].

Effective treatment for the SCLC can improve the paraneoplastic LEMS as well. For LEMS patients with SCLC, the anticancer treatment is crucial. Survival for patients with SCLC and LEMS is slightly better than for SCLC patients without LEMS [3]. However, presence of VGCC antibodies without manifest LEMS does not seem to increase survival. Nor is presence of SOX antibodies in paraneoplastic LEMS linked to any increase in survival [6].

## 8. Future Perspectives

For one group of LEMS patients, the cause of the disease is known to be a SCLC. But even if LEMS represents an early symptom of a causative tumour, the prognosis for survival is not good. More effective cancer treatment is the main challenge for this patient group. 

For the other half of LEMS patients, the cause of the disease is unknown. However, the pathogenesis is very well understood, and the pathogenic antibodies have been characterized in detail. Still the therapy is immunologically unspecific or semispecific and combined with symptomatic treatment. Antigen-specific treatment should be an aim, suppressing or modulating the immune response against VGCC specifically. The rarity of LEMS hampers research. The new and more selective immunoactive drugs already on the market and with a proven effect for less well characterized disorders, have not been tried in controlled studies for LEMS. Even uncontrolled observations are few. Multicentre evaluation for rare disorders such as LEMS is very welcomed.
